# Feeding the food insecure in Britain: learning from the 2020 COVID-19 crisis

**DOI:** 10.1007/s12571-020-01080-5

**Published:** 2020-07-13

**Authors:** Margo Barker, Jean Russell

**Affiliations:** 1 Food and Nutrition Group, Sheffield Business School, College of Business, Technology & Engineering, Sheffield Hallam University, Charles Street, Sheffield, S1 1WB UK; 2 Corporate Information and Computing Service, University of Sheffield, 10-12 Brunswick Street, Sheffield, S10 2FN UK

**Keywords:** COVID-19, Precarious working, Food banks, Charitable food, Food poverty, Food aid

## Abstract

The lockdown in Britain has rendered a large proportion of the population economically vulnerable and has at least quadrupled demand for emergency food relief. This paper looks critically at response to the crisis from the government and the voluntary sector with respect to provision of emergency food. In doing so, it has exposed gaps in understanding of the vagaries of the food supply for certain population groups and systemic weaknesses in the current system of emergency food aid. We make recommendations for healthier governmental capacity to react to a food security crisis, better relationships between the government and the voluntary sector, and further research into the dietary constraints of the precariate. Importantly, the social system needs to be responsive to short-term changes in people’s income if people are not to fall into food insecurity.

## Introduction

This paper sets out to provide an overview of the repercussions of the COVID-19 epidemic and resultant lockdown on food insecurity in Britain, highlighting the fragility of the food supply for many Britons. The official lockdown period began on 24th March 2020 and was on going at time of writing in early May.

We particularly address how food provision services for food insecure people have coped. There is a separate focus on responses from the government and voluntary sector, and an evaluation of the same. We draw on academic and think-tank reports, government press releases, accounts and blogs on websites of national institutions concerned with food poverty, posts on social media, and anecdotal information from Sheffield-based institutions with which we have established collaboration. Newspaper articles and other media reports on food poverty add further perspective to the narrative. Core to our commentary is an appraisal of the government’s response to an unprecedented acute need for food aid, juxtaposed against the experiences of the voluntary sector. The evaluation aims to identify weaknesses in response and suggest ways to mitigate these in a future crisis.

## Background

The failings of Britain’s welfare state to prevent hunger and ensure good nutrition amongst economically vulnerable people during the second decade of the twenty-first Century has been documented (Barker et al. 2019; Dowler and Lambie-Mumford 2015; Lambie-Mumford 2019; Loopstra et al. 2015). Evidence suggests that lack of financial security, including unemployment, household debt and weaknesses in the state benefits system, is a major driver of food insecurity (Davis and Baumberg Geiger 2016; Garthwaite et al. 2015; Lambie-Mumford and Loopstra 2020; May et al. 2020).

Food bank use statistics show that in 2018/19 some three million people received food parcels (Sosenko et al. 2019) compared with 41,000 in 2009/2010 (May et al. 2020). A recent tally (Independent Food Aid Network UK n.d.) estimated that the UK has about 2100 food banks, with the majority (60%) of these operated by The Trussell Trust under a social franchise model. A range of charities and community groups run the remainder; these are often reliant on volunteer labour (Loopstra et al. 2019).

Food banks source food from public donation at local collection points, surplus food donated by businesses and redistribution community schemes, as well as procurement via their own funds. Food banks largely depend on philanthropy from individuals, local businesses, charitable trusts and foundations, but also raise monies through fundraising initiatives. Exceptionally, the Trussell Trust chain has corporate sponsorship from major supermarkets, and gets income from events management and social enterprise activities (The Trustees of the Trussell Trust 2015). Food supplies are often irregular; there have been reports of shortage of some foods and excess of others, with resultant waste (May et al. 2020).

Other charitable initiatives, particularly to feed rough sleepers and other vulnerable people are current (Frost et al. 2016; Pelham-Burn et al. 2014). These include soup runs, hot meal services, community kitchens and community programmes providing children with meals during school holidays. Such frontline initiatives number over 3000 (Independent Food Aid Network UK n.d.). Anecdotal evidence suggests this is an underestimate of a diverse and fragmented branch of emergency food provision.

In short, before the current COVID-19 crisis the voluntary sector, comprising a disjointed array of charities, church organisations, not-for-profit community businesses and local community groups, has been the supplier of emergency food welfare in Britain. There is no explicit state support for people who experience acute food shortage; people often endure temporal cycles of “plenty” and “want” managing on a modicum of food of dubious nutritional quality in the lead up to a food crisis (Barker et al. 2019). The only direct state food aid operating is child-focused. However, the government’s drive to reduce food waste has offered indirect, but limited support to the charitable emergency food sector. Recently a grant aid programme under the Waste Resources Action Programme was set up to help siphon surplus food from retailers, wholesalers and manufacturers to pre-existing food redistribution charities (GOV 2019).

## Increase in food insecurity during the COVID-19 crisis

The crisis has rendered vast numbers of Britons food insecure. A large national survey estimated that there had been a quadrupling in levels of food insecurity to 16% of the population (Loopstra 2020). A short-term shortage of basic foods in the shops, inability to access shops because of self–isolation and economic reasons were the three main non-exclusive drivers of food insecurity (Loopstra 2020). Notably people from Black, Asian and minority ethnic groups, unemployed adults, households with children and people with health conditions and disability were most at risk.

The economic fall-out of the crisis and resultant lockdown has been gargantuan. Many people have therefore found themselves financially insecure. Around 1.8 million people applied for welfare support through the main benefit strut (Universal Credit) between lockdown (24th March 2020) and 4th May 2020 (BBC News 2020). Universal Credit takes five weeks to make a first payment. Moreover, 40% of Britons in employment were furloughed and sought financial support through the Job Retention Scheme (JRS) (Office for Budget Responsibility 2020). Many of the furloughed group were in low-paid employment (Office for Budget Responsibility 2020). Projections reveal that around 800,000 people working in hospitality and retail will become unemployed, while 3.1 million (46%) will have their wages reduced through furloughing (Tomlinson 2020). As two-thirds of low- and middle-income households have no savings (Tomlinson 2020), the lockdown is likely to push a substantial group of lowest paid households into acute food insecurity and further increase demand for emergency food relief.

The shutdown of the hospitality and retail sectors is pertinent because it particularly relies on workers from the gig economy (freelance employment on a per job basis often enabled through online platforms), employees on zero hours contracts (no guarantee of hours) or short hours contracts (small number of hours guaranteed) and temporary agency contracts. The growth of these groups in the labour market has been documented (MacDonald and Giazitzoglu 2019; Taylor 2017). Macdonald and Giazitzoglu cite that 1.1 million workers worked in the gig economy in 2017, while zero hours contracts made up 6% of all employment in the UK (MacDonald and Giazitzoglu 2019). This casualisation of labour has led to identification of a new group in the labour market - the precariat (Fletcher 2019; Standing 2011). The precariat describes a range of people who are employed across a medley of non-standard work patterns that are underpinned by job insecurity (MacDonald and Giazitzoglu 2019).

Many workers among the precariat, for example, gig workers are classified as self-employed, and won’t have recourse to the JRS. A small minority may be eligible for the Self-employment Income Support Scheme, but these funds were not available until June 2020 (GOV.UK 2020a). Therefore, many workers will have been dependent on Universal Credit. Equally, many people in the precariate have zero hours contracts. Before the COVID-19 crisis, people on zero hours contracts made up a significant proportion of users of independent food banks (Loopstra et al. 2019).

We contend that the precariat in general and others suffering in-work poverty (McBride et al. 2018) are particularly prone to suffer the economic effects of the crisis. While there is recourse to some government financial support, many people will be pushed further into a hand-to-mouth existence and increase need for emergency food relief.

## How the government has responded

Following closure of schools, state intervention targeted low-income families that had children eligible for free school meals. A national supermarket voucher scheme was introduced worth £15 per week per eligible child (HM Government 2020a). Schools administered the scheme and could either provide children with packed lunches or distribute the vouchers. The scheme was extended at the end of April to include families who did not usually qualify for free school meals, specifically those who had no recourse to public funds, such as migrant families (HM Government 2020b).

Unsurprisingly, given levels of food insecurity at the beginning of lockdown, the children’s voucher scheme was overwhelmed and voucher distribution was patchy (Loopstra 2020). Redemption of vouchers was not straightforward, as participating supermarkets were scarce in geographical areas with greatest social disadvantage (The Labour Party 2020). The content of the packed lunches was criticised for its quantity and quality (The Sun 2020). Such criticism of the quality of the fare offered is reminiscent of provision of nutritionally poor school food, prior to introduction of school food standards (Belderson et al. 2003; Gould et al. 2006).

Government also provided emergency funding of £3.25 m to the Waste Resources Action Programme (GOV.UK 2020b) designed to bolster food redistribution schemes during the crisis. Whilst such support may augment the food supply to food banks and other charitable providers of emergency food, the approach is piecemeal and dependent on the vagaries of commercial food supply chains. Moreover, such interventions fail to address the structural reasons why people are queuing for emergency food relief. Equally the axis with large food corporations is problematic; corporations use food waste reduction schemes to flaunt their sustainability credentials and project an altruistic corporate identity, rather than assume responsibility for their role in waste generation (Garthwaite 2019; Warshawsky 2016). Such a scheme also further institutionalises the voluntary sector, as the custodian of food security for vulnerable people.

Government emergency food aid was belatedly instigated for older people with underlying health conditions (GOV.UK 2020c). This group had been advised to self-isolate for a 4-month period. The programme was introduced after food charities reported difficulty in meeting demand (Church Action on Poverty 2020a), albeit that numerous community groups diverted operations from other food-related activities to make and deliver meals (Oliver-Larkin 2020).

There has been criticism as to the nutritional content of the food parcels destined for the shielded group; reports indicated that they lacked fruit and vegetables and were high in processed food (Butler et al. 2020). The nutritional quality of the food offer to this clinically vulnerable group is particularly pertinent. Many shielded people have co-morbidities of diabetes, cardiovascular disease and obesity, and food provision as described is likely to exacerbate these. The parcels have also been decried as inappropriate for those who may be too frail to cook, and up-scaling of the moribund meals-on-wheels service has been suggested to be a more apt intervention (Oliver-Larkin 2020).

It is clear that government was totally unprepared for this crisis. In contrast, planning to ensure food security and equitable distribution of food before the Second World War was extensive, with unprecedented emphasis on the nutritional value of food and policy intervention across all areas of the food chain (Barker and Burridge 2013).

Homeless populations are at particular risk of COVID-19; this group has a high prevalence of chronic disease and their lifestyle facilitates contagion (Tsai and Wilson 2020). At the outset of the epidemic, government initiated a relief operation to house this group; some 5500 rough sleepers were housed (GOV.UK 2020d). However, the operation stopped at housing provision. There was no endowment to cover food provision for homeless people who had been housed in self-isolation conditions.

## How the voluntary sector has responded

Trussell Trust food banks and independent food banks recorded that demand soared in the early days of the lockdown (Butler et al. 2020; The Trussell Trust 2020). Salvation Army food banks reported unprecedented demand, the number of food parcels distributed by one bank in Liverpool increased ten-fold (The Salvation Army 2020). Emergency food aid expanded as lockdown progressed. Specifically, a small independent food bank in Sheffield (“FOODHALL” n.d.) fed 252 adults and 101 children in the week commencing 20th April 2020. The corresponding figures were 30 and 4 for the week commencing 23rd March 2020 (“Mutual impact” n.d.).

The upsurge in demand for emergency food relief tested the viability of many food banks. The Independent Food Aid Network (IFAN) issued an urgent call for donations at the beginning of lockdown, and reported that the ability of many independent food banks to stay open was in doubt (Sustain 2020).

Furthermore, the restrictions of lockdown have curtailed food banks’ operations. All food banks either had to adopt a telephone- or electronic-referral system or dispense with the requirement for referral. Food banks also have had logistical difficulties in accessing food; an independent food bank in Sheffield cited frustration with an inability to book food online from supermarkets, having to shop in multiple supermarkets to obtain food stocks because supermarkets restricted sales, and a shrinking volume of donated food (Church Action on Poverty 2020b). Indeed, IFAN issued a call for banks to source food further up the supply chain to improve reliability of their food supply (Independent Food Aid Network 2020). This charity sector depends on volunteers, often retired older adults, many of whom were self-isolating. Moreover, changes to operating protocols, such as the necessity to wear personal protective equipment caused extra resource demands, and the onus to deliver food raised issues of vehicle insurance cover.

Resilience of charities is poor, especially smaller ones; these operate on a limited budget and sources of funding dried up during lockdown. Organisationally larger and more professional food banks such as the Trussell Trust were better placed. Their organisational capacity allowed development of existing partnerships with retailers, sporting bodies and food redistribution charities in the wake of lockdown, and to open new sites (Sheffield Wednesday Football Club n.d.).

The provision of food for rough sleepers in temporary accommodation largely fell on the voluntary sector services that routinely specialise in supporting this vulnerable group. Local evidence suggests that they responded similarly to food banks through forming stronger alliances between larger players, with smaller initiatives having to close.

## Conclusion

The COVID-19 crisis has brought the shortcomings of UK food welfare policy, the central role of the voluntary sector, and the major dependence of the UK on philanthropy into sharp relief. Much of the government response was piecemeal and driven by pressure groups and charities. It sought populist plasters rather than address the deep causes of food insecurity through proper planning.

This whole crisis has been exacerbated by reliance of the economy on precarious employment, especially within those sectors that were shut down. The sudden emergence of the precariat as a group vulnerable to food insecurity raises new questions about understanding the constraints that precarious working imposes on food habits, dietary inadequacies and dietary excess. The government needs to establish a framework for employment and social security that has appropriate measures to safeguard casual workers, who are particularly vulnerable to economic shocks from a pandemic or a political sea change such as Brexit.

The government’s reliance on the voluntary sector to feed the food insecure has led to a financial crisis within the charitable sector, which has been compounded by reduced donations. The government should in such emergencies be willing to provide direct funding to the voluntary sector and set up the infrastructure to do so. Equally important is the establishment of infrastructure that builds cohesive third-sector resilience, which would in an emergency allow efficient implementation of the Government’s support and ensure that people are sufficiently fed. A joined-up approach with the third sector would also enable the Government to more adequately respond to chronic food insecurity in vulnerable groups such as rough sleepers.

The crisis has highlighted how interconnected our social structures are, reinforcing the fact that no man is an island. During a pandemic we need to protect those vulnerable to food insecurity in order to protect the population as a whole. To prevent a repeat debacle, and ensure that the whole population can access sufficient resources without putting others at risk, a better understanding of the needs of the precariat is crucial. This information would enable development of a fit-for-purpose social security net.
